# Evolving random fractal Cantor superlattices for the infrared using a genetic algorithm

**DOI:** 10.1098/rsif.2015.0975

**Published:** 2016-01

**Authors:** Jeremy A. Bossard, Lan Lin, Douglas H. Werner

**Affiliations:** Department of Electrical Engineering, The Pennsylvania State University, 211A Electrical Engineering East, University Park, PA 16802, USA

**Keywords:** Cantor bar, random fractal, genetic algorithm, chaotic superlattice, infrared filter

## Abstract

Ordered and chaotic superlattices have been identified in Nature that give rise to a variety of colours reflected by the skin of various organisms. In particular, organisms such as silvery fish possess superlattices that reflect a broad range of light from the visible to the UV. Such superlattices have previously been identified as ‘chaotic’, but we propose that apparent ‘chaotic’ natural structures, which have been previously modelled as completely random structures, should have an underlying fractal geometry. Fractal geometry, often described as the geometry of Nature, can be used to mimic structures found in Nature, but deterministic fractals produce structures that are too ‘perfect’ to appear natural. Introducing variability into fractals produces structures that appear more natural. We suggest that the ‘chaotic’ (purely random) superlattices identified in Nature are more accurately modelled by multi-generator fractals. Furthermore, we introduce fractal random Cantor bars as a candidate for generating both ordered and ‘chaotic’ superlattices, such as the ones found in silvery fish. A genetic algorithm is used to evolve optimal fractal random Cantor bars with multiple generators targeting several desired optical functions in the mid-infrared and the near-infrared. We present optimized superlattices demonstrating broadband reflection as well as single and multiple pass bands in the near-infrared regime.

## 1. Introduction

Fractal geometry, which was introduced by Mandelbrot in the 1970s, has been called the ‘geometry of Nature’ because it helps us describe complex structures found in Nature that are often irregular, wiggly, self-similar on multiple length scales, and difficult to represent using conventional Euclidean geometry [1]. Fractals themselves lie at the interface between mathematics and Nature, where finite approximations to infinitely repeating fractal geometries provide a powerful mathematical tool for describing complex structures found in the natural world. Just a few of many examples of fractals in Nature include objects such as trees, rivers and shells. However, fractals have limitations in mimicking natural structures. For instance, fractal trees formed by using the same generator at every stage of growth appear too regular to have been produced by Nature [2]. Such a deterministic fractal tree would have a trunk and branches just like a normal tree, but its branches would all be identical, whereas a tree found in Nature, while possessing a self-similar shape, also has variations throughout the tree. Our eyes are able to spot the difference between the artificial structure and the legitimate item. However, if a degree of variability is introduced into the fractal generation [3,4], then the final result can appear very similar to trees found in Nature.

**Figure 1.**
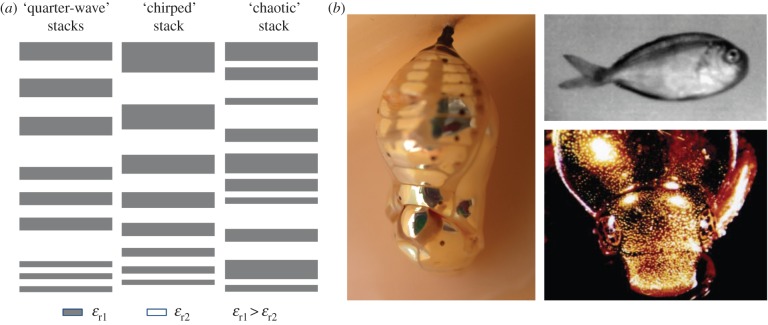
(*a*) Three ways found in Nature for achieving a broadband wavelength-independent reflector in a dielectric superlattice including three quarter-wave stacks, a ‘chirped’ stack and a ‘chaotic’ stack, inspired by Parker [5]. (*b*) Organisms with broadband optical reflectivity. (Left) Gold chrysalis of the butterfly *Euploea core* with ‘chirped’ superlattice [13]. (Top right) Ultraviolet photograph of a silvery fish with ‘chaotic’ superlattice [5]. (Bottom right) Gold beetle *Anoplognathus parvulus* with ‘chirped’ superlattice [5]. (Online version in colour.)

Superlattices, layers of homogeneous dielectric material that have contrasting refractive indices, have been identified in Nature in the skin of a variety of organisms that give rise to the spectral and polarized scattering of light off the organism [5–11]. Of particular interest is the variety of superlattice structures in Nature identified by Parker that give rise to broadband reflection, including tuned quarter-wave stacks, chirped and ‘chaotic’ multilayers, which are found in the herring, gold beetle shells and certain silvery fish, respectively [5]. Figure 1*a* illustrates these three types of superlattices found in Nature that give rise to broadband reflectivity, and photographs of several organisms with broadband reflectivity are shown in figure 1*b*. According to Parker, the first two superlattice types achieve broadband reflectivity by reflecting progressively smaller wavelengths at increasing depths within the superlattice. For instance, in the case of the tuned quarter wavelength stacks, each stack would be tuned to a different colour of light, and for the chirped superlattice, the decreasing layer thicknesses reflect decreasing wavelengths of light. In both cases, this leads to broadband reflectivity from the skin of the organism. In the third case, the superlattice for the silvery fish is characterized by Parker as ‘chaotic’ with no underlying order to the thicknesses of the lattice layers (i.e. they are modelled as purely random). However, such ‘chaotic’ geometries found in Nature may, in fact, arise from an underlying order that can be mimicked through variable fractal geometry.

Such ‘chaotic’ or disordered multi-layer reflectors are present in many animals, as indicated by the survey presented in [7]. Stacks of alternating guanine crystals and cytoplasm with random layer thicknesses occur within some silvery fish to produce broadband reflectivity [7,8], whereas similar chaotic stacks of protein platelets and cytoplasm occur within the iridophores of cephalopods [7,9]. The models proposed for these structures consist of normal or uniform distributions for the thicknesses of each material, and the number of layers typically found within the organisms [7]. Upon generating several hundred samples of chaotic stacks and averaging their reflectivity, similar broadband reflectivity was found to that which results from the actual organism [7]. While this model is able to reproduce the broadband reflectivity of the organism skin, in some cases, it ignores thicker layers of cytoplasm which are present within the skin, such as demonstrated by the ribbonfish [8].

Both these ordered and chaotic regions indicate that there is a potential design space that can be explored using fractal geometry. By introducing fractal-random structures [2], the accessible design space is extended from completely deterministic on the one hand to completely random on the other hand. For example, random fractal trees with multiple generators have been previously explored to help solve a longstanding bandwidth limitation in antenna array design [14–16]. In the past, periodic antenna arrays were typically used, because they are well understood as well as easy to model and build, but they also suffer from large grating lobes for antenna element spacings larger than a half wavelength when the main radiation beam is steered away from broadside. Completely random arrays, on the other hand, do not exhibit large grating lobes, but the sidelobe levels are unacceptably high for antenna applications and must be reduced via optimization. Random arrays are also difficult to optimize because of the large number of parameters involved in the optimization, but by employing multi-generator fractal trees to simultaneously introduce variability into the antenna array configuration and reduce the number of optimization parameters, it was feasible to synthesize large arrays using a customized genetic algorithm (GA) [14–17]. In his studies, Petko demonstrated large fractal random antenna arrays with no grating lobes and relatively low sidelobe levels for element spacings up to 20*λ*, representing a robust antenna array layout capable of operating over an ultra-wide bandwidth [15].

While multi-generator fractal trees were well suited to represent the arrangement of antenna elements in an array, a more natural choice of a fractal geometry for representing superlattices would be the Cantor bar, which is composed of one-dimensional line segments. This class of fractals has previously been studied as a basis for producing superlattices [18], where deterministic Cantor bar superlattices were found to have a characteristic, ordered structure and scattering spectra with many peaks and nulls in the reflection. However, if variability is introduced into the Cantor bar [4], then superlattices can be generated that appear less regular and more natural. Such random fractal Cantor superlattices live in the space between deterministic structures on the one hand and purely random structures on the other hand. By controlling the degree of variability in the placement of the generators, the space between the two extremes of completely ordered and completely random structures can be systematically explored. A GA is also used in order to evolve optimal fractal random Cantor superlattices with varying degrees of structure to possess specific filter functions, including a broadband reflectivity and single or multiple passbands.

## 2. Cantor bar superlattices

**Figure 2.**
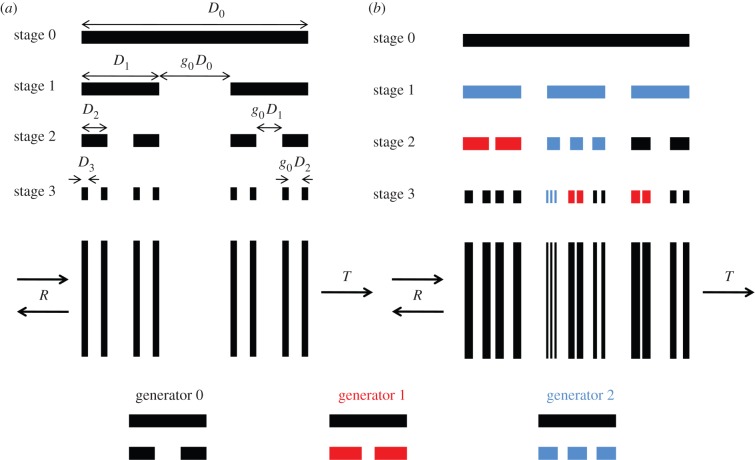
(*a*) Deterministic Cantor bar fractal and corresponding superlattice. (*b*) Random fractal Cantor bar superlattice including three generators that are randomly assigned throughout the fractal growth and corresponding superlattice. (Online version in colour.)

In 1883, Georg Cantor proposed a one-dimensional fractal that begins with a line segment from which segments are removed at every stage of growth [4]. Figure 2*a* illustrates the classic triadic Cantor bar, which has a gap size of 1/3 and deterministic growth. This one-dimensional fractal has drawn the interest of the electromagnetics community for its application to generating superlattices [18–22]. Jaggard initially formed superlattices from the triadic Cantor bar by replacing the line segments and gaps by two dielectric materials with contrasting refractive indices and simulated the scattering parameters at several stages of fractal growth [19]. He found that as the number of stages increased the scattering spectra became increasingly complex with many peaks and valleys in the reflection and transmission coefficients. Later, Jaggard & Jaggard [20] investigated the effect that increasing the number of gaps in the generator had on the superlattice scattering properties. More recently, Cantor bar superlattices have been studied as waveguides for optical light [21,22], optical filters [23,24] and light amplifiers [25].

**Figure 3.**
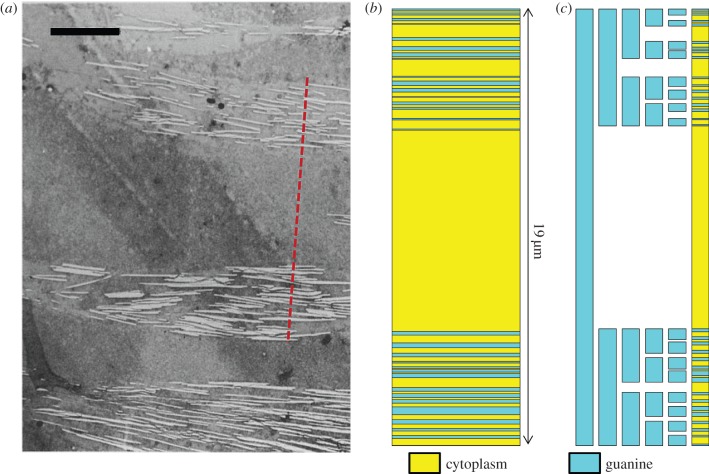
(*a*) Transmission electron microscopy (TEM) image showing a cross section of ribbonfish skin [8]. Scale bar, 5 µm. (*b*) Superlattice of cytoplasm and guanine crystal layers matching the dashed red line in (*a*). (*c*) Five-stage Cantor bar approximating the superlattice in (*b*). (Online version in colour.)

Just as deterministic fractal trees determined from a single generator do not appear to our eyes to be natural, deterministic Cantor bar fractals are also unnaturally regular in shape. In order to generate geometries that better mimic Nature, variability can be introduced into the fractal growth process. We propose to accomplish this by specifying more than one generator to be employed in the growth of the fractal geometry. Each of the generators can be assigned during the fractal growth according to a pre-specified pattern or in a random fashion. Figure 2*b* illustrates such a random fractal Cantor bar that employs three generators applied randomly throughout the fractal growth. In this illustration, the generators have a mixed number of gaps, with generators 0 and 1 inserting a single gap into the previous line segment and generator 2 inserting two gaps into the previous line segment. The fractal at stage 3 appears much less regular than its deterministic counterpart shown in figure 2*a*. A superlattice defined by the stage 3 multi-generator fractal, also shown in figure 2*b*, appears as though it could be a chaotic superlattice, such as the ones identified in the silvery fish skin. If the number and variety of generators used in the random fractal is limited, then superlattices can be produced that span the space between deterministic and random structures. For instance, if, on the one hand, only a single generator is permitted to be used in building a Cantor bar fractal, the resulting superlattice will be completely determined, whereas, at the other extreme, randomly assigning generators from an infinite pool of possible candidates would produce a superlattice that is truly chaotic. Another illustration of how a variable Cantor superlattice is well suited to represent the chaotic stack found in the ribbonfish [8] is shown in figure 3. The ribbonfish skin has several stacks of guanine crystals and cytoplasm that are separated by larger layers of cytoplasm, as shown in figure 3*a*. A 19 µm portion of this superlattice is illustrated in figure 3*b* and then approximated by a stage 5 variable Cantor superlattice with five generator gap sizes given by *g_n_* = {0.085, 0.16, 0.31, 0.46, 0.66}, where *n* indicates the generator number, and *g_n_* is multiplied by the length of the line segment to determine the gap length that will be inserted into the line segment by the generator. While the Cantor superlattice illustrated in figure 3*c* is only an approximate representation of the ribbonfish skin, it is able to capture the large lacunarity observed in the original structure. This does not imply that Nature has optimized the fish iridophore for broadband reflectance using a variable Cantor superlattice. However, the variable Cantor fractal has the capability to produce structures that appear ordered, disordered or somewhere in between, and while the approach presented here has been inspired by the ribbonfish iridophore, it is much more flexible and general such that it is not limited to only those structures that Nature can produce. Our goal in this study is to explore the space in between deterministic and chaotic superlattices by evolving random fractal Cantor bars that produce superlattices with desired spectral properties.

## 3. Genetic algorithm synthesis of random fractal Cantor superlattices

**Figure 4.**
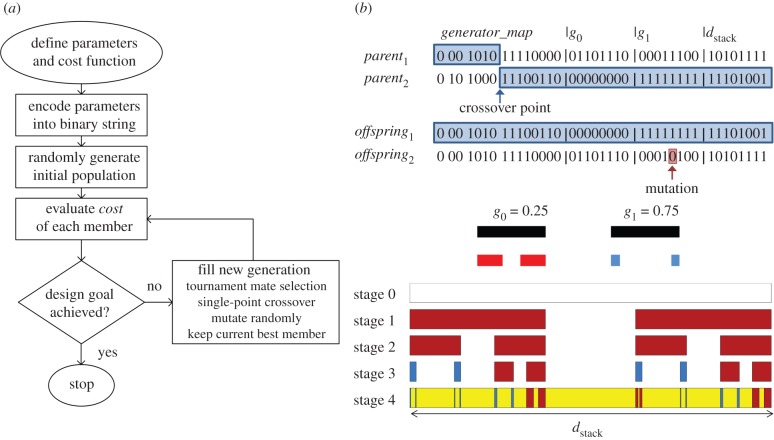
(*a*) Flowchart shows the operation of the genetic algorithm optimizer. (*b*) Illustration shows the crossover and mutation operators used to produce *offspring* from two *parent* chromosomes. The binary *offspring*_1_ chromosome has been mapped to an equivalent stage 4 multi-generator Cantor superlattice. (Online version in colour.)

In order to exploit the multi-generator Cantor bar fractal to generate superlattices with desired spectral properties, a GA was employed to evolve the superlattice structure. The GA is a robust stochastic optimizer that has been used to solve a variety of challenging electromagnetic design problems [17]. The GA is a popular optimizer within the electromagnetics community, because it is simple to implement and capable of solving problems with many design parameters. The GA itself is inspired by Nature as it emulates the natural evolutionary process, so combining it with the multi-generator fractal model allows us to not only mimic the superlattice geometries identified in Nature, but also to evolve optimum designs as would happen in Nature. The operating principle of the GA comes from the Darwinian notion of natural selection, where a population of design candidates competes for survival at each iteration of the optimization process. The operation of the GA is illustrated by a flowchart in figure 4*a*. Each population member has a binary chromosome, or a string of bits, into which the design parameters are encoded. At every iteration in the design process, all population members are evaluated for fitness and then ranked according to the individual member fitness. Members with better fitness are selected for procreation via tournament selection and then mated by crossing over their genetic data to produce two new offspring in the next generation. Crossover is accomplished by randomly selecting a point along the chromosome and then swapping the data from both chromosomes after the crossover point to produce two offspring that both contain genetic data from the two parents. A mutation operator is also applied to the new population members that randomly flips a small percentage of bits in their chromosomes, so that new regions of the parameter space are continually explored. The crossover and mutation operations are illustrated in figure 4*b*.

Just as in natural selection, the genetic data from the fittest population members are more likely to be passed on to subsequent generations, so at each iteration the GA focuses its search in the areas of the parameter space that appear more promising in terms of design performance. Mutation introduces new parameter variations that were not previously present in the design pool. Elitism is also enforced in the GA, which copies over the chromosome of the best performing member into the new generation, ensuring that the best overall performance is always maintained or improved throughout the optimization.

In order to optimize a multi-generator fractal Cantor bar superlattice, the parameters that define the Cantor bar must be encoded into the binary chromosome. For the Cantor bar, this includes the description of each generator and also the generator assignment during the growth of the fractal. If only two generators are used in the Cantor bar design, the placement can be defined by a single binary digit, where ‘0’ and ‘1’ represent generators 0 and 1, respectively. If the number of generators is increased, then the number of bits for assigning each generator must be increased accordingly. For problems with four generators, two bits are used to assign each generator. The generators themselves are defined by a single parameter indicating the gap size, *g*_n_. Bounds are placed on *g*_n_ in order to properly limit the length of the line segments or gaps in the final stage of the fractal, so that any fabrication considerations are met. For optimizations with mixed gap generators, i.e. generators with a different number of gaps, enough generator assignment bits are included in the chromosome to account for all line segments assuming that the generator with the greatest number of gaps is assigned to every node in the Cantor bar fractal. The unused generator assignment bits in a particular chromosome are ignored. The remaining parameters to optimize for the superlattice may include the permittivity of the constituent dielectric materials, the order in which the materials appear in the lattice, and the total thickness of the superlattice. Any real-valued parameters, such as the generator gap sizes, permittivities and superlattice thickness, are encoded as eight-bit strings that represent 256 increments spanning the allowed parameter ranges. The mapping of a binary chromosome, *offspring*_1_, into its equivalent four-stage multi-generator Cantor superlattice is illustrated in figure 4*b*, where the real-valued *g_n_* parameter range is {0.25, 0.75}.

The *cost* for each design is determined by first building the multi-generator Cantor superlattice from the parameters encoded in the chromosome and then calculating the reflection and transmission coefficients from the superlattice using an analytical solution for a one-dimensional dielectric stack [12]. For each optimization, a target filter function is specified in terms of *stop* and *pass* frequencies, where high reflection is desired at *stop* frequencies and high transmission is desired at *pass* frequencies. The *cost* is then calculated for each design according to the following equation:3.1
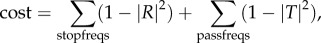
where *R* is the reflection coefficient and *T* is the transmission coefficient. As the *cost* approaches zero, the superlattice approaches perfect reflection at the *stop* frequencies and perfect transmission at the *pass* frequencies. The targeted performance was −10 dB suppression in transmission and reflection for the *stop* and *pass* frequencies, respectively.

## 4. Results and discussion

The multi-generator fractal Cantor superlattice synthesis technique presented here was developed with the objective of imitating the broadband mirror behaviour of the silvery fish skin. While the silvery fish is highly reflective over the range from UV through visible wavelengths, similar filter functions for the mid-infrared (mid-IR) and near-infrared (near-IR) regimes, as defined by the 2–10 µm and 1–2 µm wavelength ranges, respectively, were targeted where practical fabrication of the superlattices could be considered taking advantage of the refractive index contrast between deposited *a*-Si and SiO_2_ layers [26]. The first three superlattice examples presented here are broadband mirrors for the mid-IR and near-IR. Two multi-spectral examples for the near-IR follow, which show that the multi-generator fractal Cantor superlattice synthesis technique introduced, in this paper, can be extended to a variety of desired filter functions.

**Figure 5.**
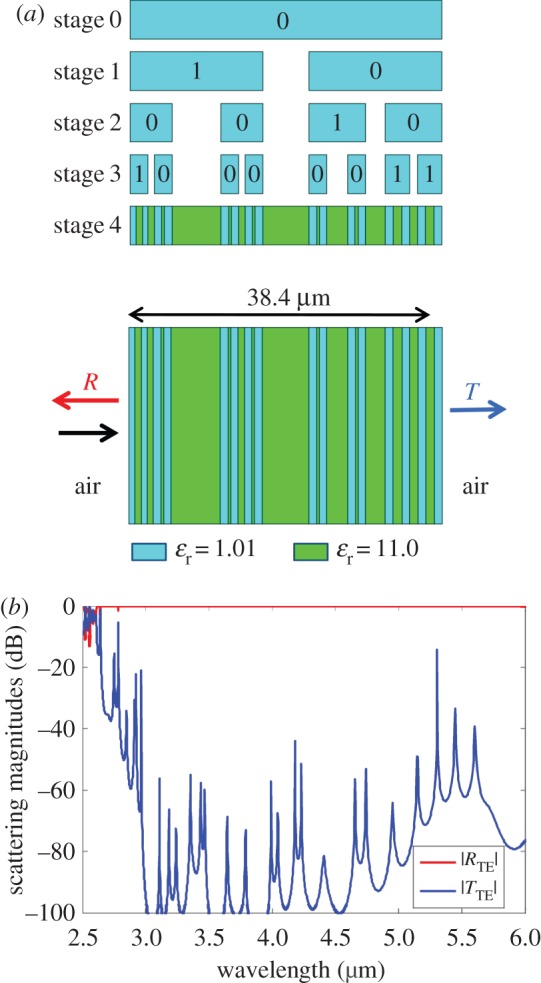
Random fractal Cantor bar superlattice with theoretical materials optimized by a GA to have broadband reflectivity in the mid-IR from 3 to 5 µm. (*a*) Random fractal Cantor bar growth indicating the generator placement and superlattice structure. (*b*) Simulated normal incidence reflection and transmission magnitudes in the mid-IR. (Online version in colour.)

In the first example, a superlattice composed of two theoretical materials is synthesized to have a broad mirror band in the mid-IR over the range from 3 to 5 µm. Forty-one *stop* frequencies were distributed uniformly over this range, so that the GA would optimize for high reflection. The GA optimized two single-gap generators and their assignments within a four-stage multi-generator Cantor bar fractal. The GA also optimized the permittivity of two theoretical materials, with the first material having a permittivity in the range from 1 to 4 and the second in the range from 5 to 11. The total superlattice thickness could range from 20 to 80 µm. The gap sizes for both generators were permitted to vary between 0.05 and 0.5. The GA optimized a population of 32 members over 1000 generations, converging on the random fractal superlattice shown in figure 5. Although the *cost* typically stopped improving around 500 generations, the GA was allowed to continue for 1000 generations to ensure that it had converged. The total thickness for the optimized superlattice is 38.4 µm, and the generator gap sizes are *g*_0_ = 0.148 and *g*_1_ = 0.369. The generator assignment is indicated in figure 5*a*. The optimized permittivities for the theoretical materials are *ɛ*_r1_ = 1.01 and *ɛ*_r2_ = 11.0, indicating that better superlattice performance was achieved by a large difference in material permittivities. The simulated reflection and transmission shown in figure 5*b* reveal that the optimized superlattice has very high reflectivity over the entire 3–5 µm range with no transmission peaks above −10 dB.

**Figure 6.**
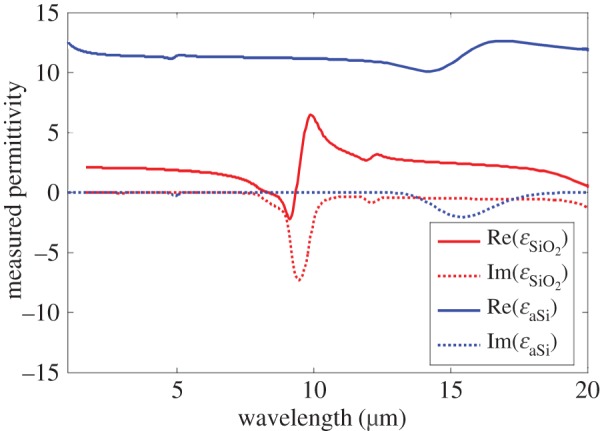
Measured dispersive permittivity for *a*-Si and SiO_2_ over the near-IR and mid-IR bands of interest. (Online version in colour.)

For the following examples, practical design parameters were imposed on the superlattice optimization. In the case of the theoretical example, the permittivities for the materials exhibited a large contrast. Thus, *a*-Si and SiO_2_ were chosen as the materials for practical superlattice designs because they have a large contrast in permittivity. Measured dispersive permittivities of *a*-Si and SiO_2_ were used in the analytical model for the superlattice and are approximately 11.6 and 2.0 in the bands of interest in the near-IR and mid-IR. Figure 6 shows the measured dispersive permittivity for *a*-Si and SiO_2_ over a wider band in the near-IR and mid-IR. These practical superlattice designs are also placed on a thick glass substrate. The intended fabrication procedure is to form the superlattice layers by iteratively depositing *a*-Si and SiO_2_. The minimum layer thickness is set to be around 20 nm.

**Figure 7.**
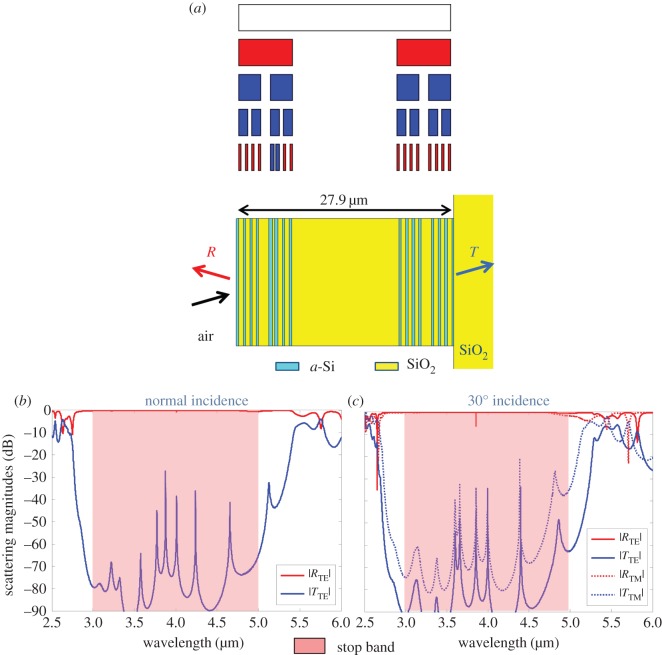
Random fractal Cantor superlattice comprised alternating *a*-Si and SiO_2_ layers on a glass substrate optimized by a GA to have broadband mid-IR reflectivity from 3 to 5 µm. (*a*) Fractal growth and superlattice structure. (*b*) Simulated scattering magnitudes at normal incidence. (*c*) Simulated scattering magnitudes at 30° off-normal incidence. (Online version in colour.)

For the first *a*-Si and SiO_2_ design, the same *cost* function was used as for the theoretical superlattice. However, the total thickness range was limited to between 5 and 40 µm, and instead of optimizing the permittivity for the two dielectric materials, the GA optimized whether the line segments in the Cantor bar would represent either *a*-Si or SiO_2_. After evolving a population of 32 members for 1000 generations, the GA converged to the multi-generator Cantor superlattice shown in figure 7*a* with a total thickness of 27.9 µm and generator gap sizes *g*_0_ = 0.495 and *g*_1_ = 0.189. In the optimized superlattice, the Cantor bar line segments are replaced by *a*-Si, and the gaps are replaced by SiO_2_. The minimum layer thickness in the structure is 220 nm, which is well above the minimum for fabrication. The simulated transmission and reflection for this design show a high reflectivity over the entire optimized band with all transmission peaks suppressed under −25 dB. Although the optimization was conducted at normal incidence, the simulated scattering curves at 30° off-normal incidence in figure 7*c* also show high reflectivity over the entire 3–5 µm band for both transverse electric (TE) and transverse magnetic (TM) polarizations, indicating that these broadband reflectors could be useful for applications requiring moderate angular insensitivity.

**Figure 8.**
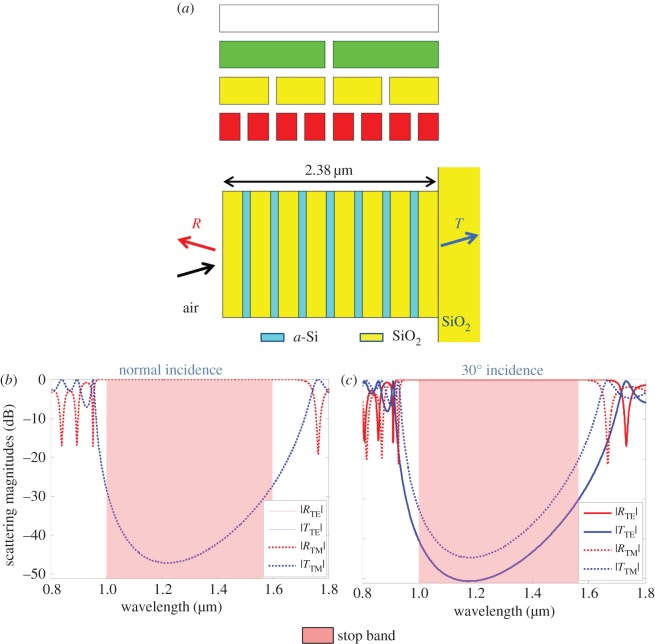
Variable Cantor superlattice optimized by a GA to have broadband near-IR reflectivity from 1 to 1.6 µm in the near-IR. (*a*) Fractal growth and superlattice structure. (*b*) Simulated scattering magnitudes at normal incidence. (*c*) Simulated scattering magnitudes at 30° off-normal incidence. (Online version in colour.)

While the broadband mid-IR mirror met the goals for fabrication, the total thickness of the superlattice and number of layers make fabrication challenging. In order to reduce the total thickness and number of layers, several adjustments were made to the optimization. In the next example, the target mirror band is moved to the near-IR band from 1 to 1.6 µm. In the *cost* function, 36 *stop* frequencies are specified covering this mirror band. The number of Cantor bar stages is also reduced to three in order to reduce the number of total layers by about half. Reducing the Cantor bar stages decreases the flexibility the GA has in varying the layer thickness in the final superlattice, so two additional generators are included in the chromosome to allow for more flexibility during the optimization. The gap size for all four generators was permitted to vary within the range from 0.02 to 0.36, whereas the total superlattice thickness could range from 0.5 to 3 µm. Once again, the GA evolved a population of 32 members for 1000 generations to produce the superlattice shown in figure 8*a*. The total thickness of 2.38 µm for this structure is much less than the previous, mid-IR examples, and the minimum layer thickness of 84 nm is well within the fabrication target. The optimized generator gap sizes are *g*_0_ = 0.161, *g*_1_ = 0.083, *g*_2_ = 0.037 and *g*_3_ = 0.075. Interestingly, only three of the four generators are assigned to nodes in the Cantor bar, indicating that the GA should be able to find a good solution with fewer generators to optimize. In the superlattice, the line segments and gaps are replaced by SiO_2_ and *a*-Si, respectively. The final optimized structure also appears approximately periodic with the *a*-Si layer thicknesses varying by 3.2%, indicating that the multi-generator Cantor bar fractal can produce geometries that span a range of appearances from nearly random to ordered. The simulated reflection and transmission spectra shown in figure 8*b* reveal that the superlattice performs well with high reflectivity over the 1–1.6 µm band for both normal and 30° off-normal incidence. The thicknesses of the *a*-Si and SiO_2_ layers are both approximately *λ*/4 within the optimized mirror band, meaning that the GA converged on a superlattice that is operating similar to a quarter-wave stack [5]. Although periodic superlattices have a characteristically narrow spectral peak, this approximately periodic structure is truncated to seven periods, which broadens the reflection bandwidth. For larger target mirror bandwidths or for larger superlattice dimensions, the GA could converge on a more disordered superlattice.

**Figure 9.**
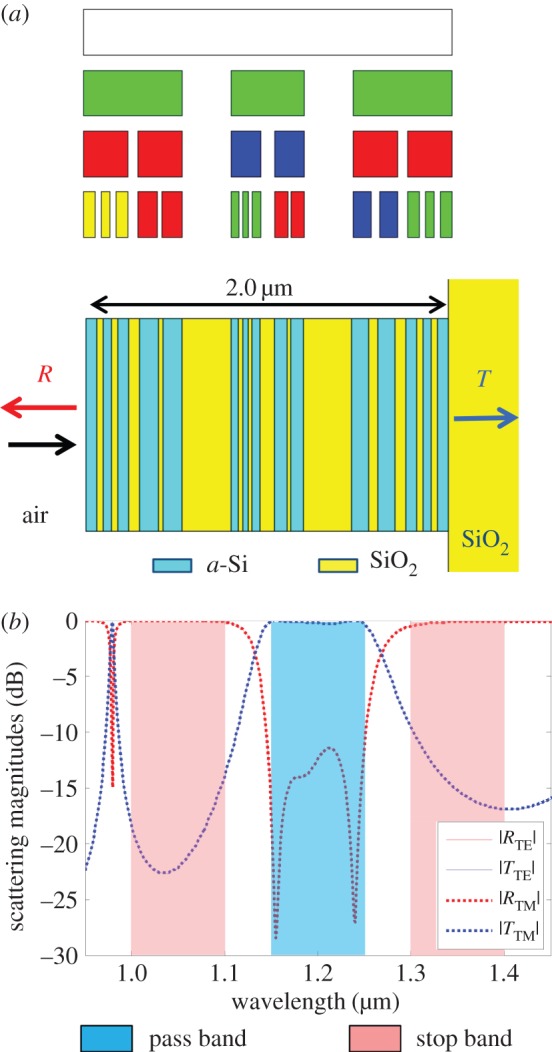
Random fractal Cantor superlattice with four mixed gap generators optimized by a GA for a single pass band surrounded by two stop bands in the near-IR. (*a*) Fractal growth and superlattice structure. (*b*) Simulated scattering magnitudes at normal incidence. (Online version in colour.)

In addition to mimicking the broadband reflectivity found in the silvery fish, the multi-generator Cantor superlattice synthesis procedure should be equally valid for other filter functions such as pass band, stop band, high/low pass and multi-spectral functions. In the next example, a filter function is targeted with a single pass band from 1.15 to 1.25 µm and stop bands on either side from 1 to 1.1 µm and from 1.3 to 1.4 µm. Six *stop* or *pass* frequencies are specified for each band in the *cost* function. For this example, the number of Cantor bar stages is again limited to three, so that the superlattice stack will have fewer layers. Four generators are optimized by the GA with a mixed number of gaps. Generators 0 and 1 introduce a single gap into the previous line segment, whereas generators 2 and 3 introduce two gaps into the previous line segment. The gap sizes for the first two generators were limited to between 0.1 and 0.2, and the gap sizes for the last two generators could vary from 0.1 to 0.15. The total thickness for the superlattice was allowed to range from 0.5 to 2 µm in the GA. The GA optimized 32 population members for 1000 generations to reach the multi-generator Cantor bar superlattice illustrated in figure 9*a*. The total thickness for this superlattice is 2.0 µm, which is at the larger edge of the allowed range. The optimized generator gap sizes are *g*_0_ = 0.107, *g*_1_ = 0.200, *g*_2_ = 0.133 and *g*_3_ = 0.145. In contrast to the previous example, this superlattice contains much more variation in layer thickness and does not possess an intuitive order, once again indicating that the GA is capable of evolving fractal Cantor bars that span the range from ordered to chaotic. This optimized superlattice replaces the line segments with *a*-Si and the gaps with SiO_2_, and the minimum layer thickness is 17.2 nm. The transmission and reflection shown in figure 9*b* have excellent pass band and stop band performance over the optimized wavelength ranges.

**Figure 10.**
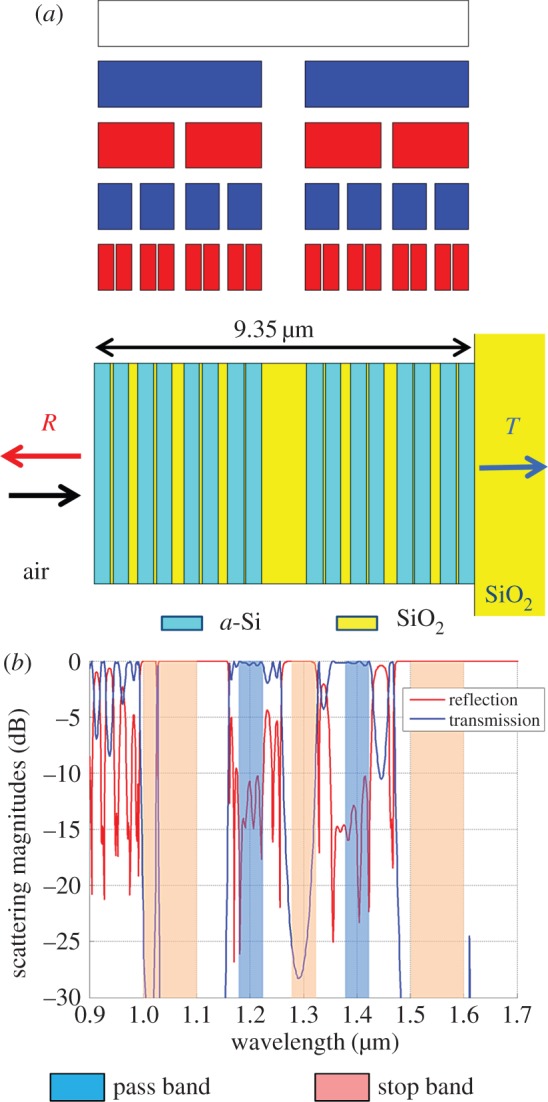
Multi-spectral random fractal Cantor superlattice optimized by a GA for two pass bands surrounded by three stop bands in the near-IR. (*a*) Fractal growth and superlattice structure. (*b*) Simulated scattering magnitudes at normal incidence. (Online version in colour.)

The final example extends the pass band filter to a multi-spectral function with two pass bands surrounded by three stop bands in the near-IR. In the *cost* function, five *pass* frequencies are specified for each of two bands from 1.18 to 1.22 µm and from 1.38 to 1.42 µm, six *stop* frequencies are specified for the bands from 1 to 1.1 µm and from 1.5 to 1.6 µm, and five *stop* frequencies are specified from 1.28 to 1.32 µm. The GA optimized a four-stage Cantor bar with two single-gap generators. The generator gap sizes could vary from 0.02 to 0.12, and the total thickness was allowed to range from 5 to 20 µm. After evolving a population of 32 members over 1000 generations, the GA converged to the superlattice shown in figure 10*a*. The optimized generator gap sizes for this Cantor bar are *g*_0_ = 0.071 and *g*_1_ = 0.119, and the total superlattice thickness is 9.35 µm. The line segments in stage 4 of the optimized Cantor bar are replaced by *a*-Si, whereas the gaps are replaced by SiO_2_. Interestingly, the optimized generator assignment is uniform for each stage, with generator 1 assigned to every bar in stages 0 and 2 and generator 0 assigned to every bar in stages 1 and 3. Hence, the superlattice shown in figure 10*a* has a somewhat regular shape similar to that of the deterministic Cantor bar. The simulated scattering coefficients show that pass bands and stop bands have the desired high transmission and reflection magnitudes, respectively. However, there is a narrow transmission peak in the shortest wavelength stop band that slipped in between the sampling frequencies. Nevertheless, this superlattice shows that the multi-generator Cantor bar can be exploited by the GA to achieve even complex filter functions.

## 5. Conclusion

While superlattices in Nature that give rise to broadband reflectivity have been classified as having various ordered and chaotic (i.e. random) structures, it is believed that the underlying order should arise from fractal geometry. Random fractals are useful for exploring the space between the ordered and chaotic extremes, because the variability in the fractal can be limited by the number and variety of generators used in its construction. We successfully employed a GA to evolve multi-generator Cantor bar superlattices that mimic the broadband filter functionality found in Nature. Among the broadband reflector and multi-spectral filter designs presented, the GA evolved structures that were highly ordered and ones that appeared chaotic. Further design flexibility of the multi-generator Cantor superlattice synthesis method was demonstrated by evolving more complex examples that had single and dual pass bands surrounded by stop bands.
